# Artificial Intelligence Estimation of Carotid-Femoral Pulse Wave Velocity using Carotid Waveform

**DOI:** 10.1038/s41598-018-19457-0

**Published:** 2018-01-17

**Authors:** Peyman Tavallali, Marianne Razavi, Niema M. Pahlevan

**Affiliations:** 1 Avicena, LLC, 2400 N Lincoln Ave, Altadena, CA 91001 USA; 2 Department of Aerospace and Mechanical Engineering, University of Southern California, Los Angeles, CA USA; 3 Division of Cardiovascular Medicine, Keck School of Medicine, University of Southern California, Los Angeles, CA USA; 4 Huntington Medical Research Institutes, Advanced Imaging Center, Pasadena, CA USA

## Abstract

In this article, we offer an artificial intelligence method to estimate the carotid-femoral Pulse Wave Velocity (*PWV*) non-invasively from one uncalibrated carotid waveform measured by tonometry and few routine clinical variables. Since the signal processing inputs to this machine learning algorithm are sensor agnostic, the presented method can accompany any medical instrument that provides a calibrated or uncalibrated carotid pressure waveform. Our results show that, for an unseen hold back test set population in the age range of 20 to 69, our model can estimate *PWV* with a Root-Mean-Square Error (RMSE) of 1.12 *m*/*sec* compared to the reference method. The results convey the fact that this model is a reliable surrogate of *PWV*. Our study also showed that estimated PWV was significantly associated with an increased risk of CVDs.

## Introduction

Cardiovascular diseases (CVDs) and stroke are among the major causes of death in the United States and the total cost related to them was more than $316 billion in 2011–2012^1,2^. New cardiovascular monitoring methods are urgently needed in order to limit the growing burden of CVDs. Arterial stiffening is one of the risk factors for CVDs^3,4^, which can be assessed non-invasively by calculating the carotid to femoral *PWV*^5^. This parameter is a gold standard of arterial stiffness, the rate at which pressure waves move down the aortic vessel^6^. Increased arterial stiffness is related to an increased risk of cardiovascular events; therefore, it has become an independent marker for CVDs^6,7^. Because of its clinical significance, there has been a surge in addressing arterial stiffness and *PWV*^8^. Arterial stiffness and its surrogates such as *PWV* have been suggested as one of the risk factors along with other biomarkers such as high cholesterol, diabetes, and left ventricular hypertrophy when cardiovascular risk is being evaluated^8^. Past studies have shown a strong correlation between *PWV* and the presence of CVDs^9–14^.

Although carotid-femoral *PWV* measurement is non-invasive, this process is intrusive as it requires the waveform collection from inguinal region. Obtaining accurate carotid-femoral *PWV* measurements often requires a well-trained staff within a clinical setting^15^. The need of the medical community is an easy-to-use and non-intrusive method to measure carotid-femoral *PWV* with acceptable accuracy and precision; see ref.^16^.

At the same time, recent advances in the field of artificial intelligence have opened up new areas and methods in creating novel modeling and predictive methods for clinical use^17^. The model and analysis in this paper are in accord to this path of introducing artificial intelligence to the field of medical sciences.

In this study, a novel, easy-to-use, and non-invasive approach to estimate carotid-femoral *PWV*, from a single carotid waveform measurement, is explored. This method is based on the newly developed Intrinsic Frequency (IF) algorithm^18,19^. IF method solely needs one uncalibrated trace of a carotid, or aortic, pressure waveform. Our method takes an uncalibrated trace of carotid pressure waveform and performs IF analysis and basic signal processing on it. Then, it combines the results of these analyses with easy-to-obtain clinical parameters such as age. Finally, it models the *PWV* by neural networks through bootstrap averaging. The main advantages in having an estimated *PWV* from an uncalibrated carotid pressure waveform, with few typical clinical variables such as blood pressure, would be that it is does not need an ECG measurement nor a femoral tonometry recording. As a result, it is easier, and potentially can be done by a smart phone as we have shown in our previous publication^20^.

## Data Description

We used the Framingham Heart Study (FHS) data, a longitudinal epidemiological cohort analysis, in this manuscript^21^. The participants were part of FHS Cohorts Gen 3 Exam 1^22^, Offspring Exam 7^23^, and Original Exam 26^24^. They underwent a comprehensive, noninvasive assessment of central hemodynamics generating a successful collection of a total of *N* = 6698 tonometry recordings. The recorded *PWV* measurements were calculated using a simultaneous right carotid tonometry pressure waveform with electrocardiogram recording and a right femoral tonometry pressure waveform with electrocardiogram recording combined with body surface measurements of the participants^7^. This method of measuring is sometimes called the sequential measurement^25^. One can find a comparison of this method to the reference method of measuring *PWV* in^25^. For a broader description regarding FHS data, please see^7^ and references contained within.

Some participants had missing or erroneous tonometry waveforms data. We marked these recordings as “faulty record” (*N* = 1011). From the rest of the data (*N* = 5687), some recorded *PWV* measurements had values equal to or greater than 30 *m*/*sec*, or even 0 *m*/*sec*. We considered these values to be “measurement error” (*N* = 21). We also excluded the population of age greater than 70 (*N* = 661) to minimize the CVD treatment effects. Also, we know that individuals having an age of 70 or greater already experience arterial stiffness due to age factor. These filterings led to a total of *N* = 5020 usable observations. Furthermore, for the prognostics study, we excluded individuals having cardiovascular diseases prior to, or on, their tonometry exam date leading to *N* = 4798 participants.

## Artificial Intelligence Methodology

### General Signal Processing

Each uncalibrated tonometry recording from the FHS data included a 10 to 20 second trace. Some of the signal processing parameters were provided by FHS data. For example the unit-less variables Augmentation Index (*AIx*) and Mean Carotid Shape Factor (*MCSF*) were included as they were provided (See^26^ and references contained within). As a quick reference, *MCSF* is the average value of an arterial cardiac cycle signal normalized by its range. Furthermore, Reflected Wave Arrival Time (*RWAT*) was also among the variables that FHS had provided with the data (See^26^ and references within).

However, to use the IF algorithm, we had to extract arterial cycles from the raw signal.

At first, a short-window moving average was used to eliminate the unwanted noise from the signals. The window size was taken to be 0.02 *sec*. This window size would eliminate noise levels above ~50 *Hz*. The blood pressure waveform can be encoded with frequencies less than ~25 *Hz* (50 *Hz* is twice this value)^27^. In mathematical terms, the moving average $\bar{s}(t)$ we used in our study, for a signal *s*(*t*), can be expressed as

1 $$\bar{s}(t)=\frac{1}{0.04}{\int }_{t-0.02}^{t+0.02}s(\tau )d\tau \mathrm{.}$$

Our method is not dependent on this choice of noise-filtering and other low-pass filtering approaches could also be used. Then the signals were normalized to remove the effects of breathing and other artificial motions. The normalization was performed using the location of maxima and minima of each recording^28^. We then used a modified version of the automatic cycle selection introduced in^29^ to pick cycles. Dicrotic notch was found based on the derivatives and filtering of the picked cycles^28^. These cycles were then fed into the IF algorithm.

### Intrinsic Frequency

A typical arterial pressure waveform consists of a systolic and diastolic part. The systolic part is when the aortic valve is open and heart is pumping blood into the aorta and arterial system. The diastolic part is when the aortic valve is closed preventing the blood from re-entering the left ventricle. The closure of the aortic valve, on the pressure waveform trace, is commonly called the dicrotic notch. The IF method^18,19^ assumes that there are two constant dominant dynamical frequencies before and after the closure of the aortic valve. These frequencies are called Intrinsic Frequencies (IFs). This method does not need a calibrated aortic or carotid pressure signal; and can even be applied to signals collected by a smart phone^20^.

In the IF method^18,19^, it is assumed that the instantaneous frequencies are piecewise constant throughout the cardiac cycle. The dicrotic notch separates these frequencies. For an aortic pressure waveform, the IF problem can be formulated as

2 $$\begin{array}{rcl}S({a}_{i},{b}_{i},\bar{p},{\omega }_{i};t) & = & ({a}_{1}\,\cos \,{\omega }_{1}t+{b}_{1}\,\sin \,{\omega }_{1}t+\bar{p}){{\bf{1}}}_{[0,{T}_{0})}(t)\\  &  & \,+\,({a}_{2}\,\cos \,{\omega }_{2}t+{b}_{2}\,\sin \,{\omega }_{2}t+\bar{p}){{\bf{1}}}_{[{T}_{0},T)}(t),\end{array}$$

with a continuity condition at *T*_0_ (the time of the dicrotic notch) and periodicity at *T* (the duration of the cardiac cycle). Here, the *indicator function* is defined as

3 $${{\bf{1}}}_{[x,y)}(t)=\{\begin{array}{cc}\mathrm{1,} & x\le t < y,\\ \mathrm{0,} & else\mathrm{.}\end{array}$$

Also, *a*_1_, *b*_1_, *a*_2_ and *b*_2_ are the envelopes of the IF model fit. *ω*_1_ and *ω*_2_ are Intrinsic Frequencies (IFs) of the waveform. Further, $\bar{p}$ is the mean pressure for the period [0, *T*). The goal of the IF model (2) is to extract a fit, called Intrinsic Mode Function (IMF), that carries most of the energy (information) from a pressure waveform *s*(*t*) in one period. The latter is done by solving the following optimization problem^19^:

4 $$\begin{array}{cc}\mathop{minimize}\limits_{{a}_{i},{b}_{i},{\omega }_{i},\bar{p}} & {\Vert s(t)-S({a}_{i},{b}_{i},\bar{p},{\omega }_{i};t)\Vert }_{2}^{2}\end{array}$$

5 $$subject\,to\,\begin{array}{ccc}{a}_{1}\,\cos \,{\omega }_{1}{T}_{0}+{b}_{1}\,\sin \,{\omega }_{1}{T}_{0} & = & {a}_{2}\,\cos \,{\omega }_{2}{T}_{0}+{b}_{2}\,\sin \,{\omega }_{2}{T}_{0},\\ {a}_{1} & = & {a}_{2}\,\cos \,{\omega }_{2}T+{b}_{2}\,\sin \,{\omega }_{2}T\mathrm{.}\end{array}$$

Here, ${\Vert \Vert }_{2}$ is the *L*^2^-norm defined on [0, *T*). One assumption in this optimization is that the extracted IMF is continuous at the dicrotic notch time *T*_0_ (the first condition in (5)). The other assumption is that the extracted IMF is periodic (the second condition in (5)). The method of the solution of the optimization problem mentioned by (4) and (5) can be found in^19^.

### Statistical Learning

Dimensionless parameters or combinatorial mixtures can be extracted from the solutions of (4) and (5). These parameters are both of mathematical and physiological importance as shown in our recent work of noninvasive iPhone measurement of left ventricular ejection^20^. For example, we can normalize *ω*_1_ and *ω*_2_ with respect to systolic and diastolic periods, *T*_0_ and *T* − *T*_0_ respectively^20^. Even, we can normalize *ω*_1_ and *ω*_2_ by the whole cardiac cycle *T*. In fact, there is a systematic way to create new variables from a set of given features. We have used the method introduced in^30^ to create new features from *ω*_1_, *ω*_2_, *T*, and *T* − *T*_0_. Some of the outputs of this method were used in our *PWV* model.

The original set of variables used for feature extraction included IFs and their variants, carotid waveform shape factors such as reflected wave arrival time and augmentation index, and clinical features and blood pressure and age. We specifically used the mentioned clinical variables since a 2010 study published by European heart journal has emphasized that *PWV* is affected by age and blood pressure^31^. In short, the original set of features is

6 $$\begin{array}{c}{V}_{0}=\{{\omega }_{1},{\omega }_{2},{\bar{\omega }}_{1},{\bar{\omega }}_{2},{\omega }_{1c},{\omega }_{2c},{\omega }_{1n},{\omega }_{2n},\rho ,{E}_{r},{C}_{r},T,\frac{1}{T-{T}_{0}},\frac{1}{{T}_{0}},\\ MCSF,AIx,SSN,RWAT,Age,{P}_{s},{P}_{d}\}\mathrm{.}\end{array}$$

In (6), we have constructed new features from {*ω*_1_, *ω*_2_, *T*, *T* − *T*_0_}. To be more specific, we used the methodology introduced in^30^ and field expert knowledge to introduce

7 $${\bar{\omega }}_{1}={\omega }_{1}{T}_{0},$$

8 $${\bar{\omega }}_{2}={\omega }_{2}\,(T-{T}_{0}),$$

9 $${\omega }_{1c}={\omega }_{1}\sqrt{{T}_{0}},$$

10 $${\omega }_{2c}={\omega }_{2}{T}^{2},$$

11 $${\omega }_{1n}={\omega }_{1}T,$$

12 $${\omega }_{2n}={\omega }_{2}T,$$

13 $$\rho =\frac{s\,({T}_{0})-\,{\rm{\min }}\,(s(t))}{{\rm{\max }}\,(s(t))-\,{\rm{\min }}\,(s(t))},$$

14 $${C}_{r}=\frac{\bar{p}-\,{\rm{\min }}\,(s(t))}{max\,(s(t))-\,{\rm{\min }}\,(s(t))},$$

15 $${E}_{r}=\frac{\sqrt{{a}_{1}^{2}+{b}_{1}^{2}}}{\sqrt{{a}_{2}^{2}+{b}_{2}^{2}}}\mathrm{.}$$

Furthermore, in (6), *MCSF* is the mean carotid shape factor, *AIx* is the augmentation index, *SSN* is the supra-sternal notch to femoral site length, *RWAT* is the reflected wave arrival time for a cardiac arterial waveform cycle, *Age* is the age of the participant at the time of tonometry reading, *P*_*s*_ is the brachial systolic pressure, and *P*_*d*_ is the brachial diastolic pressure.

In order to provide the most useful subset of these variables into the *PWV* model, we applied a combination of best subset variable selection methods based on multi-linear regression^32^. Using this approach, we ended up with the variables set

16 $$V=\{{\bar{\omega }}_{2},{\omega }_{1c},{\omega }_{1n},\rho ,{E}_{r},MCSF,AIx,SSN,RWAT,Age,{P}_{s},{P}_{d}\}\mathrm{.}$$

After this stage, we kept 20% of the data as a hold back test set (*N* = 1004). The other part was kept as a train set (*N* = 4016). These sets were picked at random from the original data. However, we chose them in a uniform way such that, both in train and test sets, the *PWV* distribution would follow the population distribution.

After this stage, on the train set, we performed a bootstrap aggregation (bagging) without replacement (sub-sampling)^33^ having single layer neural networks at the base regressors with a total number of |*V*| neurons in each network. Here, |*V*| is the number of elements in *V*. The bagging was conducted with sampling 66% at each iteration. A total number of 1000 iteration was used. It is needless to mention that one could stop the iterations when the out-of-bag RMSE reaches a plateau. At each iteration, the neural networks were trained for 100 epochs. A squared penalty of 0.01 was used to prevent over-fitting at each iteration. MATLAB implemented Levenberg-Marquardt backpropagation was used in training the neural nets^34–36^.

The whole machine learning pipeline can be expressed as follows:

1. The uncalibrated waveforms where analysed to extract cardiac cycles;
2. *IF* parameters and waveform features, such as shape factors, were extracted from the selected cardiac cycles;
3. The waveform features were blended in with the routine clinical parameters to construct the original set of features *V*_0_;
4. The best subset variable selection method was applied to reduce the dimensionality of the feature-space, namely *V*;
5. A sub-sampled bagged system of neural networks was trained and tested.

To further analyze our method and the effectiveness of estimated *PWV*, we conducted a prospective cohort study and used proportional hazards regression models to evaluate the association between *PWV* and incident CVD. We evaluated this relationship for *PWV* measurements as well as for estimated *PWV* values produced by our noninvasive IF method. Subsequently, we compared the hazards of *PWV* for CVD with that of estimated *PWV*. Baseline population consisted of participants free from CVDs. Adjusted models included components from Framingham risk score: sex, age, total cholesterol, HDL cholesterol, blood pressure, diabetes and smoking. Smoking was defined as regular usage within the last 12-months prior to the examination date. The assumption of proportionality was met. All continuous variables were log transformed to address skewness. Predictive value was evaluated via likelihood ratio test and the Akaike Information Criterion (AIC). Only complete cases without missing data were studied. Kaplan-Meier plots of cumulative probability of a first major CVD event were constructed for *PWV* and also for estimated *PWV*, when participants were grouped according to tertiles of *PWV* and tertiles of estimated *PWV*. Log rank test was used to compare the unadjusted Kaplan-Meier curves. p-values < 0.05 were considered as significant.

## Results and Discussion

### *PWV* Model Results

**Figure 1:**
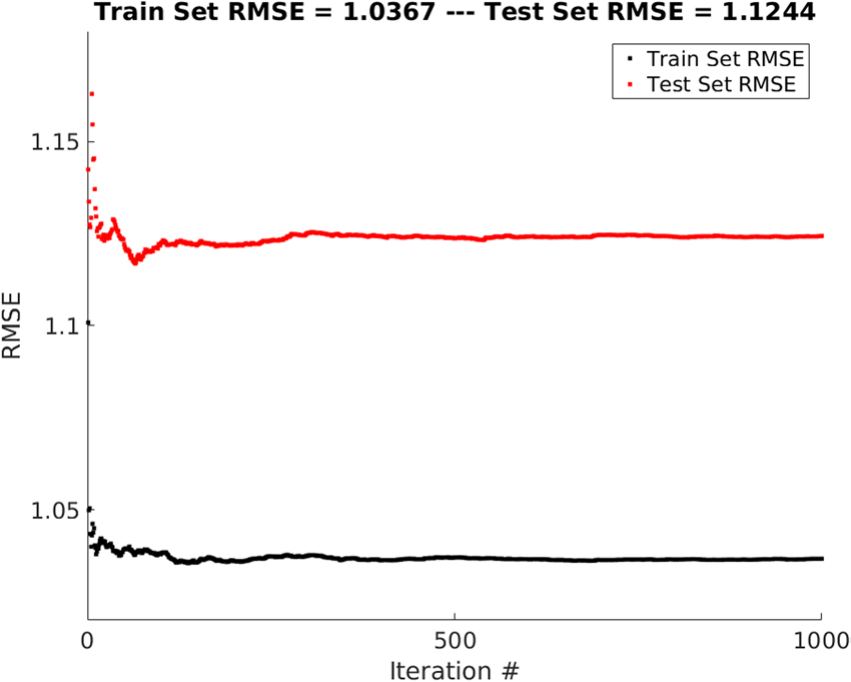
RMSE vs Iteration. The RMSE unit is *m*/*sec*. A total number of 1000 iteration is used to make sure that the ensemble of neural networks has converged. The 0.09 *m*/*sec* gap between the train and test sets shows that the model has an acceptable generalization capability.

The population demographics of these variables are shown in Table 1. Convergence of the ensemble of the models was guaranteed by a flat RMSE plot, of both train (RMSE = 1.04) and test (RMSE = 1.12) sets, over the total number of iterations, Fig. 1. The 0.09 gap between the train and test sets, in Fig. 1, shows that the model has an acceptable generalization capability. The total RMSE, on the whole dataset including the train and test sets, was 1.05 *m*/*sec*. Our simulations with Decision Tree (DT), boosted DT, boosted Neural Networks^33^ show similar but marginally larger RMSE values.

**Table 1:** Population Demographics (*N* = 5020).

Variable	Units	Mean	Standard Deviation
${\bar{\omega }}_{2}$	—	1.15	0.21
*ω*_1*c*_	$\frac{1}{\sqrt{sec}}$	51.46	2.13
*ω*_1*n*_	—	1.45	0.18
*ρ*	—	0.58	0.07
*E*_*r*_	—	2.83	0.65
*MCSF*	—	0.43	0.03
*AIx*	—	8.90	13.78
*SSN*	*mm*	536.42	39.76
*RWAT*	*msec*	134.84	25.62
*Age*	*year*	45.15	11.48
*P*_*s*_	*mmHg*	120.50	14.63
*P*_*d*_	*mmHg*	68.23	9.85
*PWV*	*m*/*sec*	7.63	2.01

**Figure 2:**
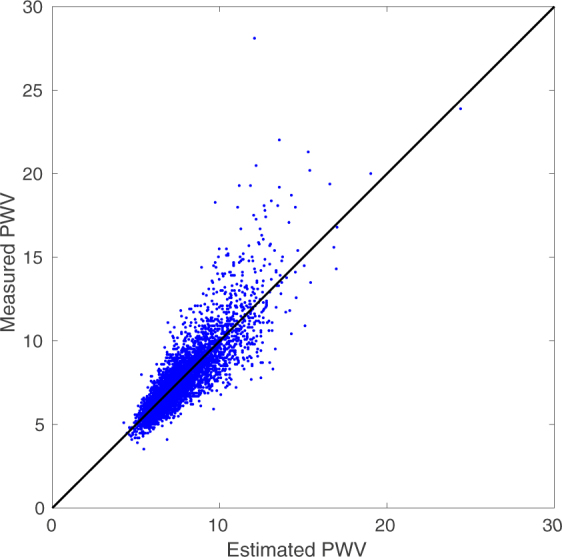
Estimated vs Measured *PWV*: The correlation is 0.85. Both axes have units of *m*/*sec*. The total RMSE is 1.05 *m*/*sec*. We can observe that as *PWV* goes above 12 *m*/*sec* the error starts to increase. For smaller values this behavior is less pronounced.

**Figure 3:**
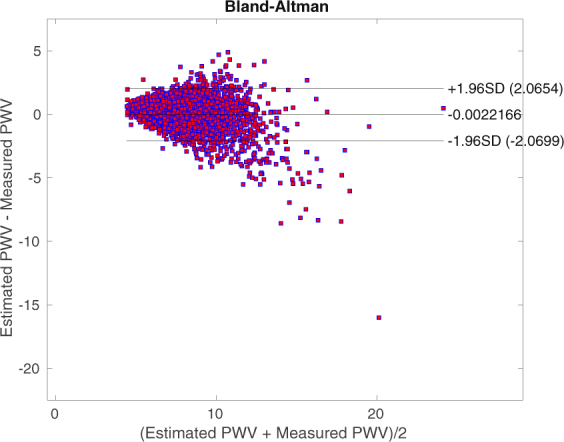
*PWV* Bland-Altman. Both axes have units of *m*/*sec*. The limits of agreement are approximately ±2.07. We can again observe that larger values of *PWV* correspond to more error.

The estimated *PWV* versus the measured values are plotted in Fig. 2. Our model’s correlation of the estimated *PWV* with respect to FHS sequential measured *PWV* is 0.85. The Bland-Altman plot of the results is shown in Fig. 3. The limits of agreement are approximately ±2.07.

### Prognosis Results

Study exclusion criteria resulted in a sample of 4798 usable observations, which included individuals 19 to 69 years old without CVD at the baseline examination. The characteristics of the study sample are presented in Table 2. Within a follow up period of 10 years, 171 participants had a CVD event. Cox proportional hazards models for *PWV* and estimated *PWV* are presented in Table 3. After adjusting for standard risk factors, both *PWV* and estimated *PWV* were significantly associated with an increased risk for a first major CVD event. Rounding for two digits after zero, the Hazard Ratio (HR) *PWV* is 4.00, with *p* = 0.0002, and the HR of the estimated *PWV* is 4.43, with *p* = 0.008.

**Table 2:** Baseline characteristics of the study population (*N* = 4798).

Characteristics	Units	Mean	Standard Deviation
*Age*	*year*	44.79	11.32
Sex, Men	%	46	—
*P*_*s*_	*mmHg*	120.34	14.53
*P*_*d*_	*mmHg*	68.1	9.8
Pulse Pressure	*mmHg*	52.25	11.18
*HR*	*bpm*	62.4	9.85
*BMI*	(*kg*)/(*m*^2^)	26.35	4.64
Total Cholesterol	(*mg*)/(*dL*)	192.54	35.56
HDL Cholesterol	(*mg*)/(*dL*)	55.47	16.49
Current smoker	%	16.3	—
Diabetes mellitus	%	3.3	—
*PWV*	*m*/*sec*	7.56	1.93
Estimated *PWV*	*m*/*sec*	7.57	1.64

**Table 3:** *PWV*and estimated *PWV* as predictors of a major CVD event (*N* = 4798). Multi-variable model adjusted for age, sex, total cholesterol, HDL cholesterol, pulse pressure, current smoking and diabetes mellitus. All continuous variables were log transformed. This table suggests that both measured *PWV* and our estimated *PWV* convey comparable risks for incident CVD in a model adjusted for standard risk factors. These results demonstrate that our estimated *PWV* is as effective as the measured *PWV*, such as sequential method, in predicting the risk for CVD.

Hemodynamic Measures	Hazard Ratio Including Standard Risk Factors	p-value
*PWV*	3.998	0.0002
Estimated *PWV*	4.43	0.008

### Model Error Analysis

We can observe, from Figs 2 and 3, that as *PWV* goes above 12 *m*/*sec* the error starts to increase. It is both because of the inherent measurement error^37–40^ and also sparsity of data in that region. For smaller values of *PWV*, this behavior is less pronounced as data concentration is higher and also the measurement errors are smaller.

We can estimate the error of our model compared to the reference method^25^ of simultaneous tonometry measurement of *PWV*. Assign the presented model output with the random variable *PWV*_*predicted*_. Also, one can label the FHS measured *PWV* values with the random variable *PWV*_*measured*_. On the other hand we can name the reference values as *PWV*_*ref*_. Based on the results of our model, the error of our model with respect to the sequential measurement of *PWV* is 1.05 *m*/*sec*. In other words,

17 $$std(PW{V}_{predicted}-PW{V}_{measured})=1.05,$$

where *std*() is the standard deviation operator. From the model results, we also have

18 $${\mathbb{E}}(PW{V}_{predicted}-PW{V}_{measured})\approx \mathrm{0,}$$

where ${\mathbb{E}}()$ is the expected value operator. Assuming that the error of the sequential *PWV* measurement is equal to the PulsePen device^41,42^, explained in^25^, we can state

19 $$std(PW{V}_{ref}-PW{V}_{measured})=\mathrm{0.31,}$$

20 $${\mathbb{E}}(PW{V}_{ref}-PW{V}_{measured})=-\mathrm{0.15.}$$

It is logical to state that the error between the reference and sequential methods is independent from the error between our model and sequential methods. As a result, using this fact and Equations (17)–(20), we can deduce that

21 $$\begin{array}{c}std(PW{V}_{predicted}-PW{V}_{ref})\\ \begin{array}{ccc}\quad =\,\sqrt{st{d}^{2}(PW{V}_{predicted}-PW{V}_{measured})+st{d}^{2}(PW{V}_{ref}-PW{V}_{measured})} & = & \mathrm{1.10,}\end{array}\end{array}$$

22 $$\begin{array}{c}{\mathbb{E}}(PW{V}_{predicted}-PW{V}_{ref})\\ \begin{array}{ccc}\quad =\,{\mathbb{E}}(PW{V}_{predicted}-PW{V}_{measured})+{\mathbb{E}}(PW{V}_{measured}-PW{V}_{ref}) & = & \mathrm{0.15.}\end{array}\end{array}$$

In other words, the estimation error, using the model presented in this paper, is approximately 1.1 *m*/*sec*. This analysis shows that our model is a reliable surrogate of *PWV*^25^.

### Comparison to other methods

Our model generated results similar to other non-invasive devices and/or methods currently in use, such as Complior^43^, PulseTrace^44^ and Oscillometric^45^.

With Complior, there is simultaneous measurement of the pressure pulse. The technician places one probe at the patient’s carotid location and a second probe at the femoral location. Then the distance between these two locations is calculated and entered into Complior software. Cuff blood pressure is also measured and entered into the software. *PWV* measures are subsequently generated after a proprietary algorithm is used to measure the pulse transit time between the two locations. With PulseTrace, the stiffness index is estimated by analyzing the photoplethysmographic waves obtained on the fingertip of the individual. The index is calculated by dividing the height of the participant by the time delay between the first systolic peak and the early diastolic peak of the signal. In a study led by Salvi *et al*. in 2008^25^, on a population of 50 participants (aged between 20–84) free from cardiac arrhythmia, these devices produced the following outputs when compared to the reference tonometry method in which waveforms were simultaneously acquired: Complior *Bias* = 2.09 *m*/*sec* and *LoA* = ±2.68 *m*/*sec*, *r* = 0.83 and PulseTrace *Bias* = −1.2 *m*/*sec*, *LoA* = ±4.92 *m*/*sec*, *r* = 0.55. Although these devices are non-invasive, they are relatively expensive and the procedure is time consuming.

In a prospectively designed validation study led by Feistrizer *et al*.^46^, aortic *PWV* estimates from oscillometric technique were generated from 40 participants free of CVDs between 24 and 55 years old. These measurements were compared to the aortic *PWV* values produced by the reference method of cardiac magnetic resonance. Analysis of agreement between the two methods showed *Bias* = 0.57 *m*/*sec*, *LoA* = ±1.92 *m*/*sec* *m*/*sec* and *r* = 0.86. In the aforementioned study, only 28% of the participants were females and the median age of the cohort was 34 years. According to the authors of the paper, the study population does not meet the Artery Society guideline’s for *PWV* validation. In specific, the Artery Society needs a homogeneous sex distribution (a minimum of 40% for either sex) as well as a homogeneous distribution along the age groups. Another study designed by Hametner *et al*.^45^, compared oscillometric estimations of aortic *PWV* against intra-aortic arterial *PWV* measurements using a population of 120 patients undergoing elective cardiac catheterization for suspected coronary artery disease (22 patients with *age* ≤ 50 years old and 29 patients with *age* ≥ 70 years old). Exclusion criteria consisted of unstable clinical conditions, arrhythmias and valvular heart disease. In their work, to estimated *PWV* a number of variables from pulse wave analysis and wave separation were combined in a mathematical model in which the major determinants were age, central pressure and aortic characteristic impedance. The following results were then reported by the authors: *Bias* = 0.43 *m*/*sec*, *LoA* = ±2.45 *m*/*sec* with a correlation of *r* = 0.81. This study also does not follow the Artery Society guidelines as only 10% of participants were females.

In a recent study by Campo *et al*.^47^, it is shown that the aortic *PWV* can be measured non-invasively with a bathroom scale. The authors combined the principles of ballistocardiography and impedance plethysmography on a single foot to estimate the aortic *PWV*. They compared their *PWV* estimations to measured *PWV* s, on a group of 205 participants. On the validation set, they reported *r* = 0.7, *Bias* = 0.25 *m*/*sec*, and *LoA* = [−2.48, 2.98]. According to the authors, this new technique presents a few limitations including the gait instability, which affects more frail elderly and some neurological diseases. Other types of diseases may also influence the applicability of the measurement, like for example atrial fibrillation or skin diseases. The population study had several exclusions too. For example, pregnant participants or participants that had morbid obesity (*BMI* > 35) were excluded.

In another recent publication^48^, Greve *et al*. argued that the need for a non-invasive *PWV* estimation is imminent because of the relative inaccessibility of devices such as high-quality applanation tonometry. They have proposed using an equation based on age and mean arterial pressure to perform the estimation. They show that in a healthy group without cardiovascular risk factors, the correlation of the estimation with measured values is *r* = 0.52. This could be seen as a major limitation of that study. Furthermore, they showed that in an apparently healthy patients with cardiovascular risk factors the correlation is *r* = 0.67. Finally, within the group with known CVDs, the correlations is reported to be very low (*r* = 0.37). They also concluded that the estimated *PWV* could predict cardiovascular events independently of the traditional cardiovascular risk factors. However,in another smaller study^49^, Greve *et al*. claimed that the estimated *PWV* only predict CVDs in apparently healthy individuals. Moreover, the estimated *PWV* reclassifies apparently healthy participants to a higher risk category.

### Innovative aspects of proposed method

The above results show that the estimated *PWV* by Intrinsic Frequencies has the potential to become a reliable non-invasive method of *PWV* measurement. Compared to Complior, PulseTrace and Oscillometric, the IF technique is less intrusive and easier to operate that offers a more practical solution to *PWV* estimations.

The presented study uses an artificial intelligence technique to render an accurate estimation of central arterial stiffness (carotid-femoral *PWV*). The sample size used in this study is large enough and includes both healthy and CVD volunteers to offer an adequate statistical power. This study also uses a homogeneous age and gender distribution population.

**Figure 4:**
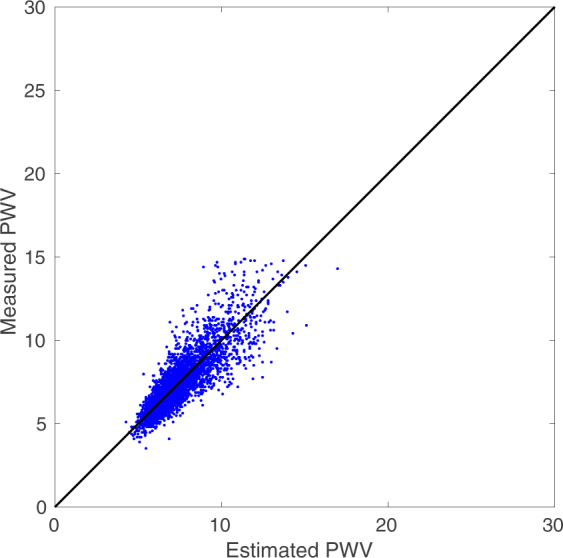
Estimated vs Measured *PWV* for *BMI* ≤ 30 and *PWV* < 15. Both axes have units of *m*/*sec*. Using the Artery Society guidelines for validation of non-invasive hemodynamic measurement devices, we observe that there is a strong agreement between our estimation of *PWV* and the recorded values.

**Figure 5:**
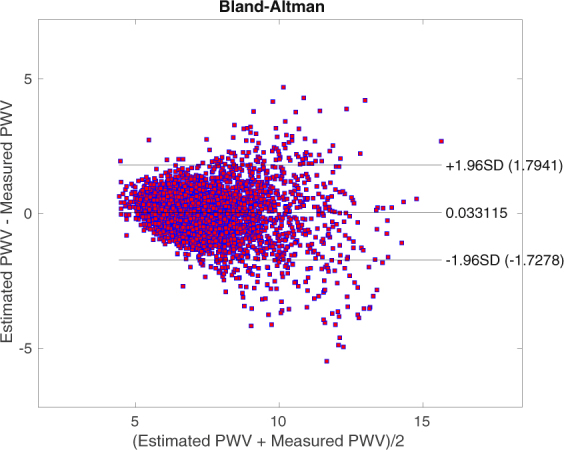
*PWV* Bland-Altman for *BMI* ≤ 30 and *PWV* < 15. Both axes have units of *m*/*sec*. This plot shows that our *PWV* estimation would be graded as acceptable by the Artery Society.

We further analyzed our results based on the Artery Society guidelines for validation of non-invasive hemodynamic measurement devices^50^. We subsequently excluded the following from our results before generating updated Bland-Altman plots: individuals with a *BMI* 30 (due to problems regarding the measurement of an accurate path length) and *PWV* ≥ 15. The updated results after the fore-mentioned filtering were: *Bias* = 0.03 *m*/*sec*, *LoA* = ±1.76 *m*/*sec* (*SD* = 0.88); see Figs 4 and 5. These outputs would be graded as acceptable by the Artery Society.

### Risk Evaluation

**Figure 6:**
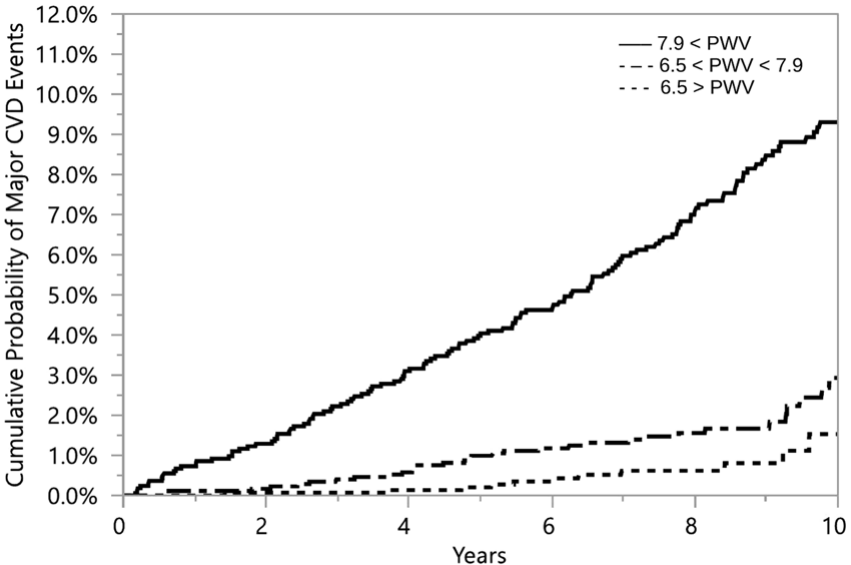
Kaplan– Meier estimates of cardiovascular disease by tertiles of *PWV* index. The solid line represents 7.9 ≤ *PWV*. The solid dashed line represents 6.5 ≤ *PWV* ≤ 7.9. The dashed line represents *PWV* ≤ 6.5.

**Figure 7:**
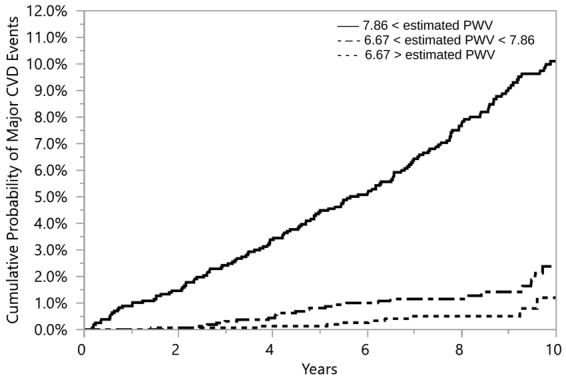
Kaplan– Meier estimates of cardiovascular disease by tertiles of estimated *PWV* index. The solid line represents 7.86 ≤ *estimated PWV*. The solid dashed line represents 6.67 ≤ *estimated PWV* ≤ 7.86. The dashed line represents *estimated PWV* ≤ 6.67.

The results, see Table 3, suggest that *PWV* and our estimated *PWV* both convey comparable risks for incident CVD in a model adjusted for standard risk factors. The use of Kaplan-Meier failure method showed that when participants were grouped according to tertiles of *PWV*, the probability of developing a CVD event increased with the group displaying higher *PWV* values (log-rank test *p* < 0.0001); see Fig. 6. The same observation was made when participants were grouped by tertiles of the estimated *PWV*. The group with estimated *PWV* of 7.86 *m*/*sec* or higher was at an increased risk of developing a CVD event (log-rank test *p* < 0.0001); see Fig. 7. These results show that estimated *PWV* by Intrinsic frequencies is as effective as *PWV* measurements obtained by direct methods, such as sequential method, in predicting the risk for CVD.

### Model Performance

**Figure 8:**
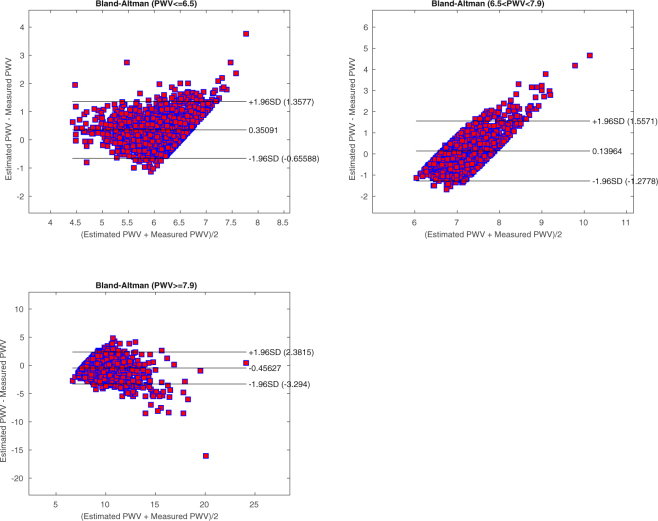
*PWV* model Bland-Altman for tertiles. All axes have units of *m*/*sec*. The tertiles are coming from the Kaplan-Meier analysis of the original *PWV* values. The upper-left plot is the Bland-Altman for *PWV* ≤ 6.5. The upper-right plot is for 6.5 < *PWV* < 7.9. The lower-left plot is the Bland-Altman for 7.9 ≤ *PWV*. All three different tertiles show that the model presented in this paper have an acceptable performance.

Following the Kaplan-Meier failure method analysis, we segmented the *PWV* data into three different groups, to check the model performance for different subsets according to *PWV* values. Figure 8 shows that in all three tertiles *PWV* ≤ 6.5, 6.5 ≤ *PWV* ≤ 7.9 and *PWV* ≤ 7.9, the model presented in this paper has an acceptable performance.

### Study Limitations

The major limitations of this study are:

1. The population used in this study was not racially diverse (mostly Caucasian). For a more general conclusion, we need to have a more diverse study population.
2. The automatic cycle selection, used in this study, is prone to mis-identification of cardiac cycles and dicrotic notches. However, the effect of the related error on overall finding of this study is insignificant.
3. The sequential method used to calculate the *PWV* might depict error either in body surface measurements of wave travel times at high *PWV* values.

## Conclusions

In this paper, we have introduced a novel artificial intelligence method to estimate the carotid-femoral pulse wave velocity. This method is based on the newly introduced Intrinsic Frequency method^19^ and as inputs uses only an uncalibrated carotid pressure waveform with typical clinical variables such as blood pressure.

The main advantages in having an estimated *PWV* from an uncalibrated carotid pressure waveform, with few typical clinical variables such as blood pressure, would be that it is does not need an ECG measurement nor a femoral tonometry recording. As a result, it is easier, and potentially can be done by a smart phone as we have shown in our previous publication that carotid waveform can be easily measured using a regular iPhone camera^20^.

Here, in this article, we have been able to address the need of estimating *PWV* by providing an accurate and precise statistical model estimating pulse wave velocity. The model presented in this manuscript can estimate *PWV* with an RMSE of 1.12 *m*/*sec*, compared to the reference method.

We have provided an error analysis and comparison to other methods currently in use in order to support the conclusion that the presented model is an acceptable surrogate for arterial stiffness. Furthermore we conducted a prospective investigation to analyze the predictive value of estimated *PWV* in relation to the onset of CVDs. Our study showed that estimated *PWV* was significantly associated with increased risk of CVDs.

### Data Availability

The data used in this study can be requested from FHS directly. It is publicly available to qualified investigators. An approved research proposals could be qualified to receive the de-identified data. FHS data, in general, can be requested by a research application submission to one of the following:

- Directly from Framingham Heart Study (https://www.framinghamheartstudy.org/), BioLINCC (https://biolincc.nhlbi.nih.gov/home/), or
- dbGaP (https://www.ncbi.nlm.nih.gov/gap).

The manuscript data can be found using the following links:

1. (https://biolincc.nhlbi.nih.gov/studies/gen3/?q=framingham) - Gen3 cohort
2. (https://biolincc.nhlbi.nih.gov/studies/framcohort/?=framingham) - Original Cohort
3. (https://biolincc.nhlbi.nih.gov/studies/framoffspring/?q=framingham) - Offspring Cohort.

### Research Ethics

All experiments and procedures were reviewed and approved by FHS. The Framingham Heart Study is conducted and supported by the National Heart Lung, and Blood Institute (NHLBI) in collaboration with Boston University (Contract No. N01- HC-25195).

### Permission to Carry Out Fieldwork

This study did not have fieldwork.
